# Arterial-Arteriolar Sclerosis Is Independently Associated With Poor Renal Outcome in IgA Nephropathy Patients

**DOI:** 10.3389/fmed.2021.761897

**Published:** 2021-11-17

**Authors:** Lingqiu Dong, Jiaxing Tan, Fangming Li, Siqing Wang, Zheng Jiang, Aiya Qin, Zhengxia Zhong, Xiaoyuan Zhou, Yi Tang, Wei Qin

**Affiliations:** ^1^West China School of Medicine, Sichuan University, Chengdu, China; ^2^Division of Nephrology, Department of Medicine, West China Hospital of Sichuan University, Chengdu, China; ^3^Division of Nephrology, Department of Medicine, Chengdu Seventh People's Hospital, Chengdu, China; ^4^Division of Nephrology, Department of Medicine, Affiliated Hospital of Zunyi Medical College, Zunyi, China; ^5^West China School of Public Health, West China Forth Hospital of Sichuan University, Chengdu, China

**Keywords:** IgA nephropathy, end-stage renal disease, renal survival, chronic kidney disease, arterial-arteriolar sclerosis

## Abstract

**Aim:** This study aimed to investigate the clinicopathological features and prognosis of immunoglobulin A nephropathy (IgAN) with arterial-arteriolar sclerosis (AS).

**Methods:** Patients with biopsy-proven IgAN from the West China Hospital of Sichuan University were retrospectively enrolled. Clinicopathological features were collected. Patients were categorized based on the presence and the severity of the AS. All the patients were regularly followed-up until a composite end point. The correlation between AS and prognosis of IgAN was assessed.

**Results:** A total of 1,424 patients were recruited and followed for 60.0 ± 28.7 months. Patients with AS tended to have older age, higher blood pressure, heavier proteinuria, higher serum creatinine, uric acid, and total triglyceride (TG). Meanwhile, they were more likely to have a lower estimated glomerular filtration rate (eGFR), hemoglobin, and albumin. At the end of follow-up, 126 patients in the AS group and 47 patients in the non-AS group had reached the composite end point (*p* < 0.001). AS was associated with the renal outcome (log-rank *p* < 0.001) and was an independent risk factor for the progression of IgAN (*p* = 0.049). The severity of AS was associated with renal outcomes (log-rank *p* < 0.001) and there was a trend that it might serve as an independent risk marker for progression of IgAN. In the subgroup analysis, patients presenting with AS and lower eGFR, albumin, and hemoglobin or higher proteinuria, uric acid, and TG had a significant trend for a shorter time to reach the end point (log-rank *p* < 0.001).

**Conclusion:** AS was commonly seen in patients with IgAN and was independently associated with the poor prognosis.

## Introduction

Immunoglobulin A nephropathy (IgAN) is the most common primary chronic glomerular disease globally (1). It is characterized by the predominance of IgA deposits in the mesangium and requires renal biopsy for diagnosis (2). The pathological features of IgAN on light microscopy vary significantly among patients and can be reflected in the variable clinical features (3). The Oxford classification of IgAN has identified five widely validated factors: mesangial hypercellularity (M), endocapillary hypercellularity (E), segmental sclerosis (S), interstitial fibrosis/tubular atrophy (T), and crescents (C) to have independent value in predicting renal outcomes (4, 5). Besides these parameters, intrarenal arterial and arteriolar lesions, including thickening of the intimal wall and hyaline, can also be observed commonly in 40–55% of patients with IgAN (6–9). Intrarenal arteries and arterioles are responsible for supply of oxygen and nutrients, the lesions of which may lead to chronic hypoxia and renal injury (10). However, its effect on the progression of IgAN is still controversial and its relationship with other clinicopathological factors remains unclear. This retrospective study mainly focuses on arterial-arteriolar sclerosis (AS) and its relation with the renal outcome and other identified risk factors for poor kidney prognosis.

## Methods

### Participants

From February 2009 to September 2017, a total of 1,545 renal biopsy-proven patients with IgAN from the West China Hospital of Sichuan University were evaluated for this retrospective study. Diagnosis of IgAN was based on the Kidney Disease: Improving Global Outcomes (KDIGO) Clinical Practice Guidelines for Glomerulonephritis (11). Patients with systemic lupus erythematosus (SLE), Henoch–Schönlein purpura (HSP), diabetes, liver disease, or malignancy were not included in this study. After 30 patients with incomplete data, 14 patients with a follow-up < 1 year and 77 patients with fewer than 8 glomeruli in the renal biopsy slides were excluded; the remaining 1,424 patients were included in this study. This study was conducted following the Declaration of Helsinki principles and was approved by the Medical Ethical Committee of West China Medical School, Sichuan University (FF-33-2019). Written informed consent was obtained from all the patients.

### Renal Histopathology

Renal biopsies were examined by immunofluorescence microscopy, light microscopy, and electron microscopy. H&E stain, Masson's trichrome stain, periodic acid-Schiff (PAS) stain, and periodic acid silver methenamine (PASM) stain were performed to assess the pathological features and AS. The lesions were reviewed by an experienced pathologist and a nephrologist according to the Oxford classification of IgAN (MEST-C scores): mesangial score ≤ 0.5 (M0) or >0.5 (M1); segmental glomerulosclerosis absent (S0) or present (S1); endocapillary hypercellularity absent (E0) or present (E1); tubular atrophy/interstitial fibrosis < 25% (T0), 26–50% (T1), or >50% (T2); and crescents absent (C0), <25% (C1), ≥25% (C2). AS was defined as the presence of arterial intimal thickening or arterial-arteriolar hyaline changes in efferent arterioles, afferent arterioles, artery, and arterioles in the interstitium. Furthermore, intimal thickening was scored 0–2 according to the Oxford classification (12): score 0, no intimal thickening is present; score 1, intimal thickening less than the thickness of the media; and score 2, intimal thickening more than the thickness of the media. Hyaline change was assessed as presence or absence. Microangiopathy (MA) was defined as the presence of thrombi, endothelial swelling or denudation, intramural fibrin, or intimal swelling in the small artery and/or arterioles.

### Clinical Data Collections

The baseline was established at the time of kidney biopsy. We systematically recorded sex, age, systolic blood pressure, diastolic blood pressure, and laboratory data including serum creatinine (SCr), estimated glomerular filtration rate (eGFR), hemoglobin (Hb), serum uric acid (UA), serum albumin (ALB), total serum cholesterol (TC), serum triglyceride (TG), 24-h proteinuria, and hematuria. eGFR was calculated according to the Chronic Kidney Disease Epidemiology Collaboration (CKD-EPI) formula. Anemia was defined as Hb < 120 g/L in male or Hb < 110 g/L in female. Hyperuricemia was defined as UA > 420 μmol/L in males or UA > 360 μmol/L in females. Persistent hematuria was defined as urinary red blood cell more than 5 per high power field (HPF).

### Treatment

Medications during follow-up were also collected. All the patients received optimized supportive treatment with full-dose angiotensin-converting-enzyme inhibitor (ACEI) or angiotensin receptor blockers (ARBs) and blood pressure was controlled below 140/90 mm Hg. Corticosteroids therapy (0.5–1 mg/kg prednisone daily, tapering down within 6–8 months) were used in patients with persistent proteinuria (protein excretion > 1 g/d) and receiving a maximum dose of ACEI/ARB. Immunosuppressant therapy (cyclophosphamide 2 mg/kg daily for 3 months or mycophenolate mofetil 1–2 g daily for 6–8 months) was used based on the pathological classification and clinical severity.

### Outcomes

The primary end point was a composite end point defined as a ≥50% reduction in eGFR, end-stage renal disease (ESRD), or death. ESRD was defined as eGFR ≤ 15 mL/min/1.73 m^2^ or the requirement for renal transplantation or maintenance dialysis.

### Statistics Analysis

Normally distributed variables were presented as mean ± SD and compared by using the Student's *t*-test. Non-normally distributed variables were expressed as median with interquartile ranges and analyzed with the Mann–Whitney *U* test. Categorical variables were described as the frequency with percentage and compared with the chi-squared test and the Fisher's exact test. The Kaplan–Meier method was used to analyze the survival probability between AS groups and other subgroups and survival curves were compared with the log-rank test. The univariate and multivariate Cox proportional hazards regression analysis were performed to explore the influence of variables on the composite end point. The relationship between AS and other variables was assessed by the logistic regression. A two-tailed *p* < 0.05 was considered as statistically significant. All the statistical analysis was performed with the Statistical Package for the Social Sciences (SPSS) statistics version 26.0 software (IBM Corporation, Armonk, New York, USA).

## Results

### Demographic and Clinical Characteristics

A total of 1,424 biopsy-proven patients with IgAN from the West China Hospital of Sichuan University were enrolled in this retrospective study. Demographic, clinical, and pathological characteristics of included patients are shown in Table 1. Patients were followed for 60.0 ± 28.7 months. All the patients were categorized according to the presence of AS. Among all the 1,424 patients, 674 patients (310 males and 364 females) presented AS, while 750 patients (330 males and 420 females) presented non-AS. Besides, 187 patients (12.7%) also had hyaline changes. The mean age of patients in the AS group was 36.9 ± 11.2 years and the mean age of patients in the non-AS group was 32.1 ± 10.5 years. Compared with the non-AS group, patients presenting with the AS group had more tendency to have higher systolic blood pressure (SBP) and diastolic blood pressure (DBP) (SBP: 132.1 ± 19.3 vs. 123.4 ± 16.0, *p* < 0.001; DBP: 86.6 ± 14.4 vs. 80.1 ± 11.5, *p* < 0.001) and hypertension (281 vs. 124, *p* < 0.001). Laboratory findings showed that patients with AS had more proteinuria [1.86 (1.00–3.00) vs. 1.09 (0.64–2.35), *p* < 0.001], higher serum creatinine [96.0 (73.0–130.7) vs. 75.0 (61.4–50.7), *p* < 0.001], serum uric acid [387.5 (316.0–460.25) vs. 348.5 (285.2–418.7), *p* < 0.001], and total TG (1.99 ± 1.31 vs. 1.72 ± 1.34, *p* < 0.001); however, they had lower eGFR (77.1 ± 31.5 vs. 101.3 ± 30.2, *p* < 0.001), Hb (130.8 ± 21.0 vs. 134.6 ± 18.8, *p* < 0.001), and albumin (38.7 ± 6.13 vs. 39.4 ± 6.82, *p* = 0.046). With respect to treatment, a large proportion of patients with AS received immunosuppression and/or steroids (23.0 vs. 18.1%, *p* < 0.001) and they were less likely to be treated with supportive treatment (38.0 vs. 46.7%, *p* < 0.001) only.

**Table 1:** Characteristics of immunoglobulin A nephropathy (IgAN) patients with or without arterial-arteriolar sclerosis (AS).

	**All**	**Non-AS**	**AS**	***P*-value**
	**(*n* = 1,424)**	**(*n* = 750)**	**(*n* = 674)**	
**Clinical features**				
Age, y	34.4 ± 11.1	32.1 ± 10.5	36.9 ± 11.2	<0.001
Sex (male)	640 (44.9)	330 (44.0)	310 (46.0)	0.45
Hypertension, %	405 (28.4)	124 (16.5)	281 (41.7)	<0.001
Proteinuria > 1 g/d	970 (68.1)	452 (63.3)	518 (76.9)	<0.001
Persistent hematuria, %	1,166 (81.9)	638 (85.1)	528 (78.3)	0.001
SCr, μmol/L	84.0 (65.2–110.0)	75.0 (61.4–05.7)	96.0 (73.0–130.7)	<0.001
eGFR, mL/min/1.73 m^2^	89.8 ± 33.1	101.3 ± 30.2	77.1 ± 31.5	<0.001
eGFR <60 mL/min/1.73 m^2^, %	304 (21.3)	84 (11.2)	220 (32.6)	<0.001
Anemia, %	195 (13.7)	75 (10.0)	120 (17.8)	<0.001
Uric acid, μmol/L	374.4 (301.0–442.0)	348.5 (285.2–418.7)	387.5 (316.0–460.25)	<0.001
Hyperuricemia, %	596 (41.9)	257 (34.3)	339 (50.4)	<0.001
Albumin, g/L	39.0 ± 6.51	39.4 ± 6.82	38.7 ± 6.13	0.046
TC, mmol/L	5.04 ± 1.52	5.02 ± 1.63	5.06 ± 1.40	0.59
TG, mmol/L	1.85 ± 1.33	1.72 ± 1.34	1.99 ± 1.31	<0.001
**CKD stage, %**				
Stage 1	751 (52.7)	504 (67.2)	247 (36.6)	<0.001
Stage 2	369 (25.9)	162 (21.6)	207 (30.7)	<0.001
Stage 3	248 (17.4)	76 (10.1)	172 (25.5)	<0.001
Stage 4	52 (3.7)	8 (1.1)	44 (6.5)	<0.001
Stage 5	4 (0.3)	0 (0)	4 (0.6)	0.35
**Pathologic features, %**				
M1	1,075 (75.5)	503 (67.1)	572 (84.9)	<0.001
E1	67 (4.7)	24 (3.2)	43 (6.4)	0.005
S1	861 (60.5)	401 (53.5)	460 (68.2)	<0.001
T1-T2	294 (20.6)	60 (8.0)	234 (34.7)	<0.001
C0/C1-C2	325 (22.8)	142 (18.9)	183 (27.2)	<0.001
**Medications, %**				
Supportive treatment	606 (42.6)	350 (46.7)	256 (38.0)	<0.001
Steroids only	527 (37.0)	264 (35.2)	263 (39.0)	0.138
Immunosuppression and/or steroids	291 (20.4)	136 (18.1)	155 (23.0)	<0.001
**Follow-up**				
Duration, mon	60.0 ± 28.7	62.4 ± 29.1	57.2 ± 28.1	0.001
Composite endpoint, %	173 (12.1)	47 (6.3)	126 (18.7)	<0.001

*Values presented as number (percentage), mean ± SD, or median (interquartile range)*.

*SCr, serum creatinine; eGFR, estimated glomerular filtration rate; TC, total cholesterol; TG, triglyceride; M1, mesangial hypercellularity; E1, endocapillary hypercellularity; S1, segmental glomerulosclerosis; T1–T2, tubular atrophy/interstitial fibrosis > 25%; C1-C2, crescents > 0*.

### Histopathological Characteristics

**Figure 1:**
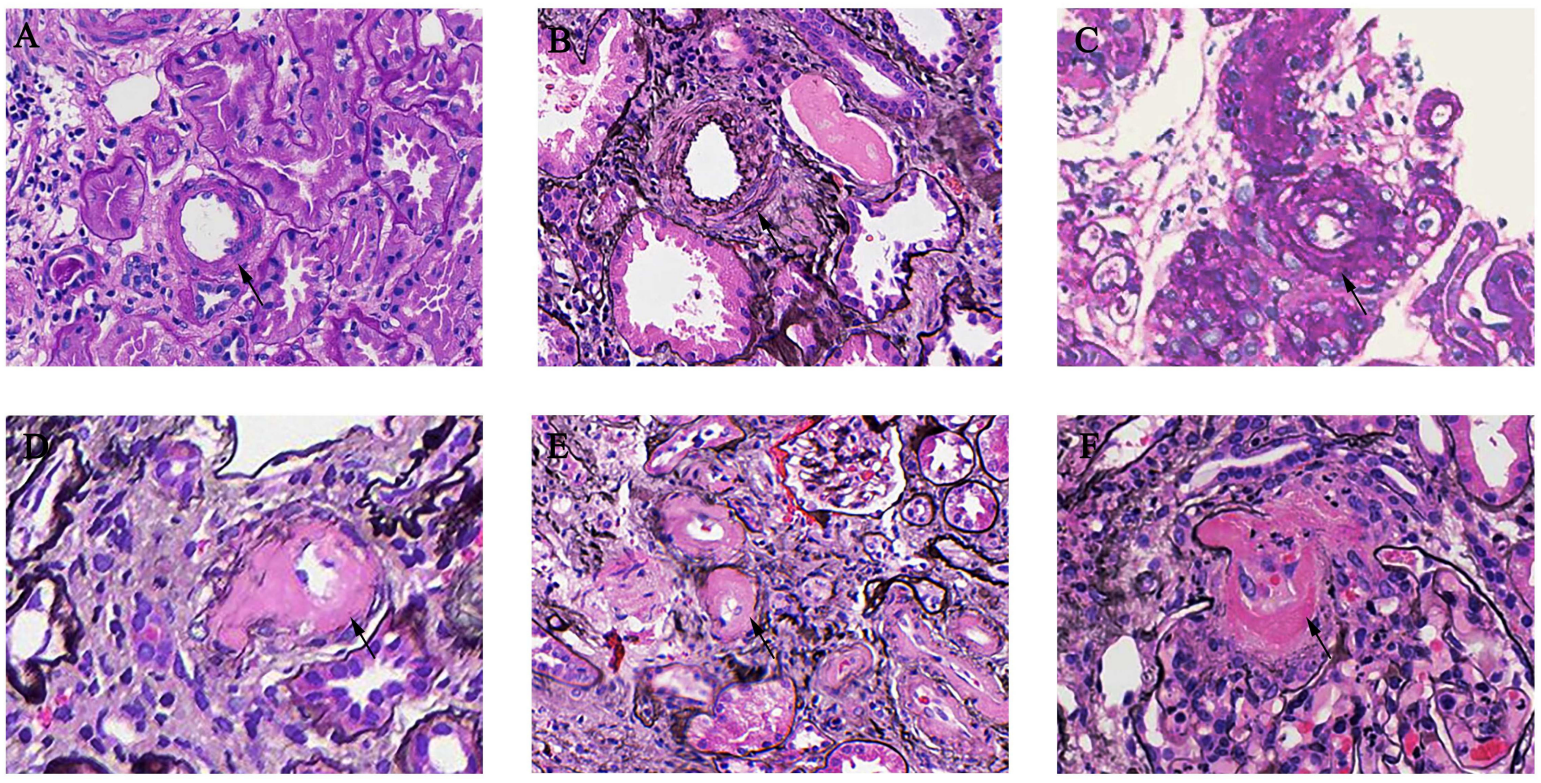
Arterial-arteriolar lesions in immunoglobulin A nephropathy (IgAN). **(A)** Normal artery [periodic acid-Schiff (PAS) stain]. **(B)** Arteries with intimal thickening less than the thickness of the media [periodic acid silver methenamine (PASM) stain]. **(C)** Arteries with intimal thickening more than the thickness of the media (PASM stain). **(D)** Arteries with hyaline changes (PAS stain). **(E)** Arteries with hyaline changes (PASM stain). **(F)** Microangiopathic lesions (PASM stain).

As shown in Table 1, mesangial hypercellularity was found in 572 (84.9%) patients in the AS group compared with 503 (67.1%) patients with the non-AS group (*p* < 0.001). There was also a significant trend of greater prevalence of endocapillary hypercellularity (6.4 vs. 3.2%, *p* = 0.005), segmental glomerulosclerosis (68.2 vs. 53.5%, *p* < 0.001), tubular atrophy/interstitial fibrosis (34.7 vs. 8.0%, *p* < 0.001), and crescents (27.2 vs. 18.9%, *p* < 0.001) among patients with AS compared to patients with non-AS. In our cohort, 74 patients (5.20%) presented with MA. Endothelial swelling was observed in 71 patients (4.99%) and 5 patients (0.35%) with thrombi. Different arterial-arteriolar lesions were shown in Figure 1.

### Outcomes

At the end of the follow-up, a total of 173 patients (12.1%) had reached the composite end point. As shown in Table 1, there were 126 patients (18.7%) in the AS group progressed to composite end point compared with 47 patients (6.3%) in the non-AS group (*p* < 0.001).

**Figure 2:**
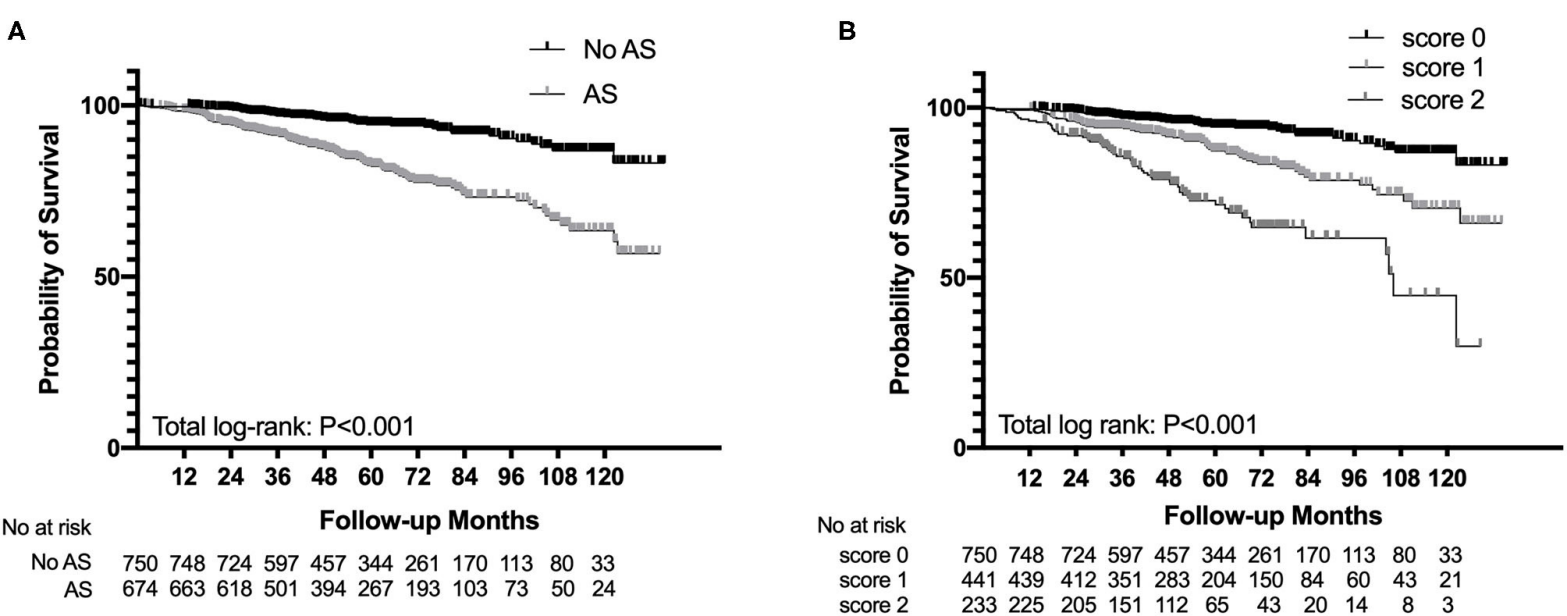
Survival probability from end-stage renal disease (ESRD), a 50% reduction of estimated glomerular filtration rate (eGFR), or death in patients with IgAN with the presence of arterial-arteriolar sclerosis (AS) **(A)** and the severity of intimal thickening **(B)**.

The Kaplan–Meier analysis revealed that AS was significantly associated with poor renal outcome and the survival rate of patients in the AS group was much lower than that of patients in the non-AS group (log-rank *p* < 0.001, Figure 2A). The severity of AS was associated with renal outcomes. Patients with intimal thickening less than the thickness of the media (score 1) had a lower survival rate than patients without AS (score 0), meanwhile patients with intimal thickening more than the thickness of the media (score 2) had the worst renal outcome among three groups (log-rank *p* < 0.001, Figure 2B). However, there was no difference in composite end point between the hyaline changes groups (log-rank *p* = 0.91) and the MA groups (log-rank *p* = 0.110).

**Figure 3:**
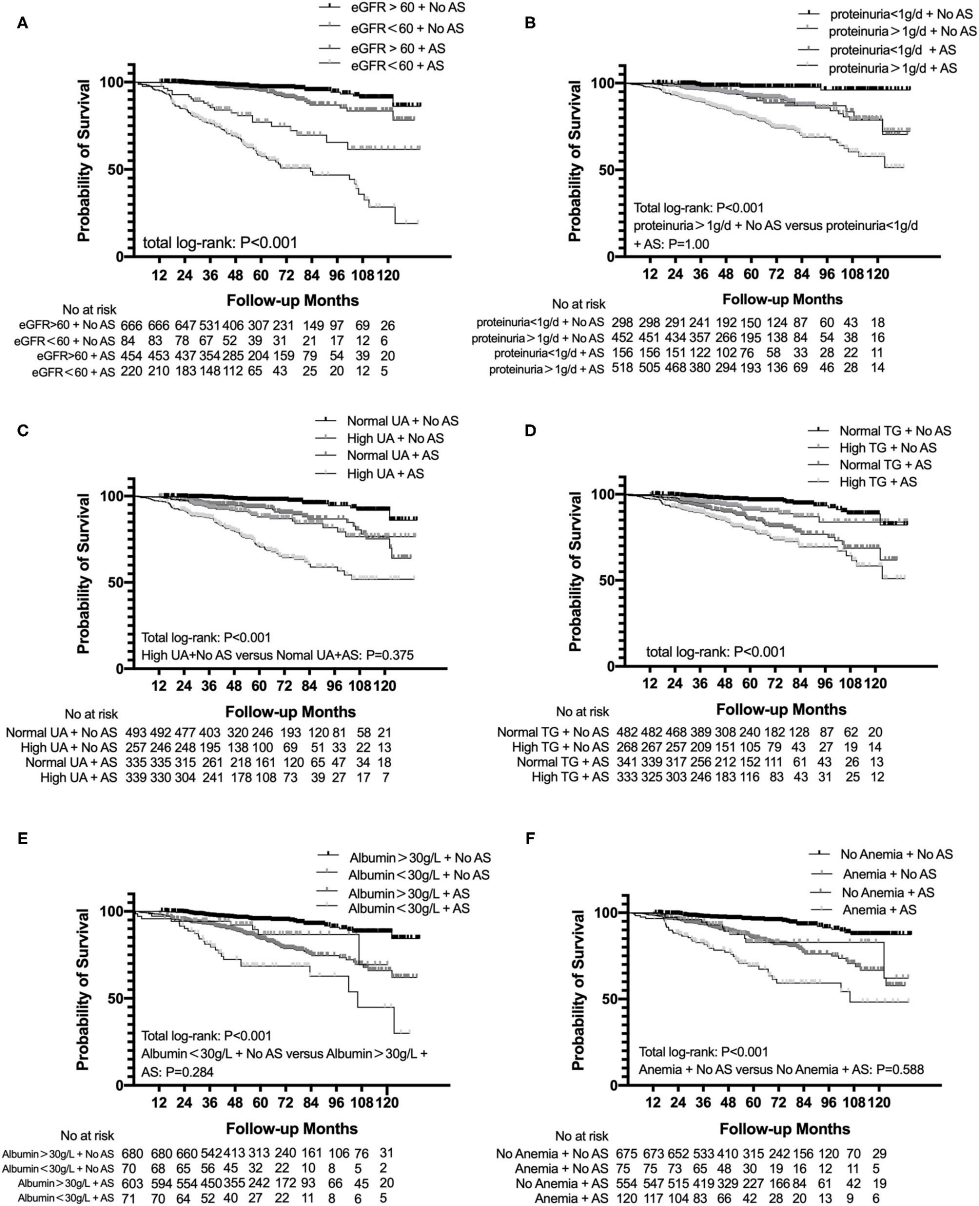
Subgroup analysis for survival probability from ESRD, a 50% reduction of eGFR, or death in patients with IgAN with AS and eGFR **(A)**, proteinuria **(B)**, uric acid **(C)**, total triglyceride **(D)**, albumin **(E)**, and hemoglobin **(F)** levels. eGFR, estimated glomerular filtration rate; UA, uric acid; TG, triglyceride.

The subgroup analysis categorized based on eGFR < 60 ml/min/1.73 m^2^, proteinuria > 1 g/d, albumin < 30 g/l, anemia, uric acid, and TG suggested that patients both presenting with AS and eGFR < 60 ml/min/1.73 m^2^ had a significant trend for a shorter time to reach end point than other subgroups. Similar results were found in proteinuria > 1 g/d (*p* < 0.001), albumin < 30 g/l (*p* < 0.001), anemia (*p* < 0.001), uric acid (*p* < 0.001), and TG (*p* < 0.001) subgroups (Figure 3).

The Cox proportional hazards regression analysis was performed to explore the risk factor of the progression of IgAN. The univariate analysis showed that AS was significantly associated with a higher risk of the incidence of the composite end point [hazards ratio (HR) = 3.327, 95% CI: 2.379–4.653, *p* < 0.001]. As shown in Table 2, three models were used for the multivariate Cox proportional hazards regression analysis. After adjusting for age, gender, the Oxford classification of IgAN (MEST-C scores), MA, eGFR, proteinuria, hematuria, hyperuricemia, anemia, TG, ALB, and treatments (model 3), AS was an independent risk factor of the progression of IgAN to the composite end point (HR = 1.451, 95 CI%: 1.002–2.103, *p* = 0.049). In addition, age, segmental glomerulosclerosis, tubular atrophy/interstitial fibrosis, proteinuria, eGFR, hyperuricemia, albumin, and treatment were also significantly related to the development of composite end point. Besides, there seemed to be a trend that the severity of AS might serve as an independent risk marker for progression of IgAN, though the difference was not statistically significant in model 3 of the Cox proportional hazards regression analysis (score 0 vs. score 1: *p* = 0.052, score 0 vs. score 2: *p* = 0.135). These results may indicate that single-grade classification (presence and absence) may be a better indicator for renal outcomes than semi-quantification methods (score 0–2).

**Table 2:** The Cox proportional hazards regression models for composite endpoint in patients with IgAN.

**Variable**	**Univariant**		**Model 1**		**Model 2**		**Model 3**	
	**HR (95% CI)**	***P***	**HR (95% CI)**	***P***	**HR (95% CI)**	***P***	**HR (95% CI)**	***P***
AS	3,327 (2.379–4.653)	<0.001	1.738 (1.206–2.505)	0.003	1.986 (1.396–2.826)	<0.001	1.451 (1.002–2.103)	0.049

*Model 1 adjusted age, gender, Oxford classification of IgA (MEST-C scores), and microangiopathy*.

*Model 2 adjusted age, gender, eGFR, proteinuria, hematuria, hyperuricemia, anemia, total TG, albumin, and treatments (supportive treatment, steroids only, and immunosuppression and/or steroids)*.

*Model 3 adjusted covariates in model 1 and model 2*.

### Relationship Between AS and Clinicopathological Features

As shown in Table 3, the logistic regression analysis was conducted to analyze the relationship between AS and clinicopathological features. It revealed that the existence of mesangial hypercellularity [odds ratio (OR) = 2.143, 95% CI: 1.596–2.879, *p* < 0.001], tubular atrophy/interstitial fibrosis (OR = 3.76, 95% CI: 2.623–5.389, *p* < 0.001), and crescents (OR = 1.433, 95% CI: 1.072–1.915, *p* = 0.015) were significantly related to the presence of AS. Notably, proteinuria more than 1 g per 24 h (OR = 1.477, 95% CI: 1.123–1.943, *p* = 0.005), older than 30 years old (OR = 1.844, 95% CI: 1.427–2.383, *p* < 0.001), and hypertension (OR = 2.275, 95% CI: 1.718–3.014, *p* < 0.001) were also strongly suggested a tendency of the presence of AS.

**Table 3:** The logistic regression models for the presence of AS.

**Variable**	**OR**	**95% CI**	***P*-value**
M	2.143	1.596–2.879	<0.001
E	1.564	0.876–2.794	0.131
S	1.237	0.961–1.592	0.099
T0/T1-T2	3.76	2.623–5.389	<0.001
C0/C1-C2	1.433	1.072–1.915	0.015
Proteinuria > 1 g/d	1.477	1.123–1.943	0.005
eGFR < 60 mL/min/1.73 m^2^	1.326	0.934–1.881	0.114
Hyperuricemia	0.98	0.755–1.273	0.88
Hyperlipemia	1.277	0.990–1.648	0.06
Hypercholesteremia	0.77	0.587–1.011	0.059
Anemia	1.08	0.746–1.566	0.682
Hypoalbuminemia	1.111	0.716–1.724	0.638
>30 years old	1.844	1.427–2.383	<0.001
Hypertension	2.275	1.718–3.014	<0.001

*OR, odds ratio; eGFR, estimated glomerular filtration rate; M1, mesangial hypercellularity; E1, endocapillary hypercellularity; S1, segmental glomerulosclerosis; T1–T2, tubular atrophy/interstitial fibrosis > 25%; C1-C2, crescents > 0*.

## Discussion

In this retrospective study, AS was found in 43.7% of the whole cohort, similar to the Oxford study (4) and a Chinese cohort established by the Peking University (6). Moreover, we also found that AS had a significant influence on the progression of IgAN. The probability of a ≥50% reduction in eGFR, ESRD, or death during the follow-up was significantly higher in the AS group than patients with the non-AS group. The presence of AS was still an independent factor for the progression of the disease after adjusting for both the clinical and pathological variables, meanwhile the severity of AS did not show the same results, which indicated that the single-grade classification of AS might be a better indicator of the prognosis of IgAN than semi-quantification.

However, the effect of AS on the progression of IgAN was controversial in the previous studies. Russo et al. reported that patients with arterial diseases were at a higher risk of progression to death or ESRD compared to patients without it and patients having both the arterial disease and high uric acid showed a shorter time progressing to end point compared to those having only one risk factor, which was consistent with this study (7). Moreover, as Zhang et al. reported in a retrospective study, they also found that the presence of vascular lesions was associated with poorer renal outcomes (9). However, in the original Oxford study, arterial and arteriolar lesions were evaluated as artery score, which showed that arterial and arteriolar lesions were not associated with the rate of renal function decline. A similar conclusion also has been drawn by Cai et al. (6). Artery score has not been discussed in the further Oxford study (5). The possible causes for this inconsistency may arise from the several aspects. In this study, most of our patients were resident in the Southwest of China and the other cohort mentioned above mainly originated from Caucasian or Northern China, different populations among different cohorts were considered as one important reason. Also, the baseline characteristics were different among cohorts. There were more patients with severe pathological classifications and heavier proteinuria in our cohorts, which can result in the different treatment methods proportion between the studies. Furthermore, renal AS is considered as a chronic lesion and its effects on renal survival also may not reflect thoroughly in a short time of follow-up. Therefore, long-time follow-up was needed to explore the relationship between AS and renal outcomes. This study had a comparatively larger sample size and a longer follow-up duration than other studies; therefore, the duration of follow-up may also partly explain the inconsistent conclusions.

In our center, 74 patients (5.20%) presented with MA. Endothelial swelling was observed in 71 patients (4.99%) and only 5 patients (0.35%) had thrombi. However, there was no difference in composite end point between the MA groups (log-rank *p* = 0.110). This may be due to the low prevalence of MA in our cohort.

In this study, patients with AS tended to be older and have higher blood pressure, heavier proteinuria, a higher level of serum creatinine, serum uric acid, and total TG. Meanwhile, they were more likely to have a lower eGFR, Hb, and albumin. Also, in the logistic regression analysis, the existence of mesangial hypercellularity, tubular atrophy/interstitial fibrosis, crescents, persistent proteinuria, older age, and uncontrolled hypertension before diagnosis were also strongly suggested a higher tendency of the presence of AS. These results were similar to recently published studies (6–9). Among all these variables, most of the variables were reported to be risk factors of the progression of the disease including mesangial hypercellularity, tubular atrophy/interstitial fibrosis, crescents, eGFR, persistent proteinuria, anemia, total TG, and high level of uric acid (13–17). This was also confirmed in the subgroup analysis that eGFR, persistent proteinuria, high uric acid, TG, reduced Hb, and albumin have a synergic effect with AS on renal outcomes. It is well-known that hypertension and total TG were closely related to the pathogenesis of arterial sclerosis (18, 19). However, AS also has been observed in patients without hypertension. In this study, we confirmed that 58.3% of AS did not have hypertension. This suggested that hypertension may not be the only reason that leads to AS. Moreover, in a recently published study, a high level of uric acid seemed to have a synergic effect with the arterial lesions on the renal outcome, caused by the proliferation of vascular smooth muscle cell activated by uric acid through mitogen-activated protein kinases and stimulating cyclooxygenase-2 and platelet-derived growth factor (GF) synthesis (7, 20). With respect to eGFR and persistent proteinuria, these factors were already identified to be strong prediction factor of the poor renal outcome for patients with IgAN in many former large-scale studies, which have been already applied as instructions for treatment in the KDIGO guidelines (11). Serum albumin is closely related to urinary protein excretion and also it was reported as an independent risk factor of prognosis of IgAN (21). Anemia and AS may also have a synergistic effect on chronic renal hypoxia and contribute to the progression of the disease (22).

To evaluate the effect of different variables on the progression of the disease, the multivariate Cox proportional hazards regression analysis was performed and both the age, gender, clinical variables, pathological variables, and medications were all taken into account. Notably, all the hypertension cases in our cohort were carefully treated with antihypertensive therapy and the blood pressure reached targeted blood pressure (within 140/90 mm Hg) during the follow-up. The multivariate Cox proportional hazards regression analysis confirmed that AS was an independent risk factor for renal progression of patients with IgAN.

The possible mechanism for AS contributing to the progression of the disease may be hypoxia. It was reported that arteriolar hyalinosis was a possible surrogate marker of reduced interstitial blood flow and hypoxia in glomerulonephritis (23). AS leads to the impairment of arterial and arteriolar functions on offering oxygen and nutrients to tubular and interstitial cells, resulting in chronic ischemia of the tubular cells and interstitium, together with the distortion and loss of peritubular capillaries (24). Hypoxia is a profibrogenic factor for tubular cell and accelerates interstitial fibrosis, which can largely reduce oxygen diffusion efficiency, and then forms a vicious circle (25). Hypoxia can also lead to podocyte injury by overexpressing hypoxia inducible factor-1 (HIF1). Podocytes can present with podocyte epithelial-mesenchymal transition (EMT), slit-diaphragm dysfunction, foot process effacement, and cytoskeletal derangement due to accumulation of HIF1, which contributed to pathology of glomerular diseases and proteinuria (26).

This study has some limitations that deserve to be mentioned. Firstly, patients recruited in this study were mainly from the Southwest China and this study is a retrospective single-center study. The baseline between the two groups was not entirely balanced, which was a common limitation of this retrospective study. We performed multivariate analysis to eliminate the confounding factors, but this study may still not suitable for all the regions of the world. Secondly, the follow-up duration was relatively short compared to the progressive speed of IgAN. Thus, the findings still need further confirmation.

## Conclusion

Arterial-arteriolar sclerosis was commonly seen in patients with IgAN and was independently associated with the poor prognosis.

## Data Availability Statement

The raw data supporting the conclusions of this article will be made available by the authors, without undue reservation.

## Ethics Statement

The studies involving human participants were reviewed and approved by the Medical Ethical Committee of West China Medical School, Sichuan University. The patients/participants provided their written informed consent to participate in this study.

## Author Contributions

LD: design of the work, analysis and interpretation of data, and drafting and revising the manuscript. JT and SW: analysis and interpretation of data. FL: acquisition and analysis of data. AQ and ZJ: analysis of data. ZZ and XZ: acquisition of data. YT: conception and supervision of the work. WQ: conception and design of the work. All authors involved in the final approval of the manuscript to be published.

## Funding

This study was partly supported by the National Key R&D Program of China (2020YFC2006503) and grants from the project of the National Natural Science Foundation of China (No. 81970612).

## Conflict of Interest

The authors declare that the research was conducted in the absence of any commercial or financial relationships that could be construed as a potential conflict of interest.

## Publisher's Note

All claims expressed in this article are solely those of the authors and do not necessarily represent those of their affiliated organizations, or those of the publisher, the editors and the reviewers. Any product that may be evaluated in this article, or claim that may be made by its manufacturer, is not guaranteed or endorsed by the publisher.
